# 

**Published:** 1828-11

**Authors:** 


**TO SUBSCRIBERS.**—For want of space we have been obliged to omit the entire department of Miscellaneous Intelligence.

The Editor regrets that the infirm state of his hands from the severe burns received in September, should still be a cause of delay in the publication of the Journal. He is happy to say, however, that the December number will appear by the end of that month.

